# Comparing the transcriptome of developing native and iPSC-derived mouse retinae by single cell RNA sequencing

**DOI:** 10.1038/s41598-023-28429-y

**Published:** 2023-01-21

**Authors:** Anouk Georges, Arnaud Lavergne, Michiko Mandai, Fanny Lepiemme, Latifa Karim, Loic Demeulenaere, Diego Aguilar, Michael Schyns, Laurent Nguyen, Jean-Marie Rakic, Masayo Takahashi, Michel Georges, Haruko Takeda

**Affiliations:** 1 GIGA Stem Cells, GIGA Institute, University of Liège, Liège, Belgium; 2 Department of Ophthalmology, Faculty of Medicine and CHU University Hospital, University of Liège, Liège, Belgium; 3 GIGA Bioinformatics Platform, GIGA Institute, University of Liège, Liège, Belgium; 4 Laboratory for Retinal Regeneration, Center for Developmental Biology, RIKEN, Kobe, Japan; 5 GIGA Genomics Platform, GIGA Institute, University of Liège, Liège, Belgium; 6 Unit of Animal Genomics, GIGA Institute, University of Liège, Liège, Belgium; 7 Digital Business, HEC Management School, University of Liège, Liège, Belgium; 8 Laboratory for Retinal Regeneration, Center for Biosystems Dynamics Research, RIKEN, Kobe, Japan

**Keywords:** Developmental biology, Stem cells

## Abstract

We report the generation and analysis of single-cell RNA-Seq data (> 38,000 cells) from mouse native retinae and induced pluripotent stem cell (iPSC)-derived retinal organoids at four matched stages of development spanning the emergence of the major retinal cell types. We combine information from temporal sampling, visualization of 3D UMAP manifolds, pseudo-time and RNA velocity analyses, to show that iPSC-derived 3D retinal organoids broadly recapitulate the native developmental trajectories. However, we observe relaxation of spatial and temporal transcriptome control, premature emergence and dominance of photoreceptor precursor cells, and susceptibility of dynamically regulated pathways and transcription factors to culture conditions in retinal organoids. We demonstrate that genes causing human retinopathies are enriched in cell-type specifying genes and identify a subset of disease-causing genes with expression profiles that are highly conserved between human retinae and murine retinal organoids. This study provides a resource to the community that will be useful to assess and further improve protocols for ex vivo recapitulation and study of retinal development.

## Introduction

It has recently become possible to produce organoids that recapitulate morphological and functional features of the native retina from pluripotent stem cells (iPSCs) in human and mice^1–3^. This has opened new avenues to explore the molecular mechanisms underlying retinogenesis and differentiation of each of the major cell types during retinal neurogenesis. It offers hope to improve therapies for retinal degenerative diseases which afflict tens of millions of people in the US and Europe alone and may account for approximately 50% of all cases of blindness^4^. Stem cells derived from somatic cells of patients offer new opportunities to study the effects of gene defects on human retinal development in vitro and to test small molecules or biologics to treat the corresponding disorders^5,6^.

Assessing how faithfully retinal organoids recapitulate specific developmental programs has typically been done by monitoring the expression of limited numbers of cell-type specific markers and examining the spatial patterning of the corresponding groups of cells^7^. Interrogating the expression of a handful of marker genes/proteins does not fully inform about the proper temporal and spatial execution of the epigenetic program, nor the presence of minor aberrant cell types. Single-cell RNA-sequencing (scRNA-Seq) now enables profiling of the transcriptome of individual cells. This permits the clustering of cells based on the similarity of their transcriptome and the identification of cellular subtypes including some that may not have been recognized before^8–11^. It also allows to refine developmental trajectories by identifying cells occupying intermediate states connecting clusters in multidimensional expression space^12,13^ and by predicting the developmental orientation taken by individual cells based on measured deviations from the steady-state ratio between spliced and unspliced RNA molecules (“RNA velocity”)^14,15^. Genes that are defining cellular sub-types can be pinpointed by differential expression analysis between clusters^16^, while genes that drive the differentiation process may be identified by searching for gene sets that are dynamically regulated across real and/or pseudo-time^17^.

Recently, scRNA-Seq has been used to compare transcriptome dynamics during native and embryonic stem cells (ESC)- or iPSC-derived retinal development in human^18–21^. This has confirmed comparable cellular compositions and convergent cell-type specific transcriptomes at equivalent developmental time-points with, however, some notable differences in gene expression as well as structural differences including disrupted inner retinal lamination in advanced organoid stages compared with human fetal retina^21^. It has revealed previously unrecognized cell type- and stage-specific expression of genes underpinning inherited diseases (such as Leber congenital amaurosis, retinitis pigmentosa, stationary night blindness and achromatopsia), that was largely recapitulated in retinal organoids^19^.

In mice, transcriptome analysis by scRNA-Seq has been reported for developing native retinae, but not yet for retinal organoids^8–11^. One of the advantages of mouse retinal organoids as biological models is the speed with which these reach developmental maturity (~ 30 days versus ~ 210 days for human retinal organoids)^1,19^. Transplantation of mouse iPSC-derived retinal tissue to mice with retinal degeneration showed partial restoration of retinal structure and function^22–25^.

Here we report the generation and use of scRNA-Seq data collected at four matched stages of retinal development in native retinae and iPSC-derived retinal organoids in the mouse to study the dynamics of the transcriptome and compare it between the two systems. This is the first report of a scRNA-Seq dataset for developing mouse retinal organoids, which remain an essential tool to study retinal development, disease pathogenesis, drug discovery, and regenerative therapies.

## Results

### Joint analysis of scRNA-Seq data from native retinae and iPSC-derived retinal organoids highlights canonical cell types and developmental trajectories

To contribute to the comparison of the developmental trajectories in native retinae (NaR) and iPSC-derived 3D retinal organoids (RO), we performed scRNA-Seq of murine NaR and RO at four matched stages of development: embryonic day (E)13 vs differentiation day (DD)13, postnatal day (P)0 vs DD21, P5 vs DD25, and P9 vs DD29^22^. NaR were dissected from 2 to 12 C57BL/6 mice per stage. Mouse RO were generated from the Nrl-GFP (C57BL/6 background) iPSC line^26^ following^22,27^ (Fig. S1). Optic vesicle-like structures (OV) were manually dissected from average ten RO per stage. Cells from NaR and OV were dissociated and subjected to droplet-based scRNA-Seq using a 10× Genomics Chromium platform. We obtained sequence information for 21,249 cells from NaR and 16,842 cells from RO, distributed evenly amongst developmental stages. We generated an average of 74,808 reads per cell, corresponding to 5940 unique molecular identifiers (UMIs) and 2471 genes per cell (Table S1).

**Figure 1:**
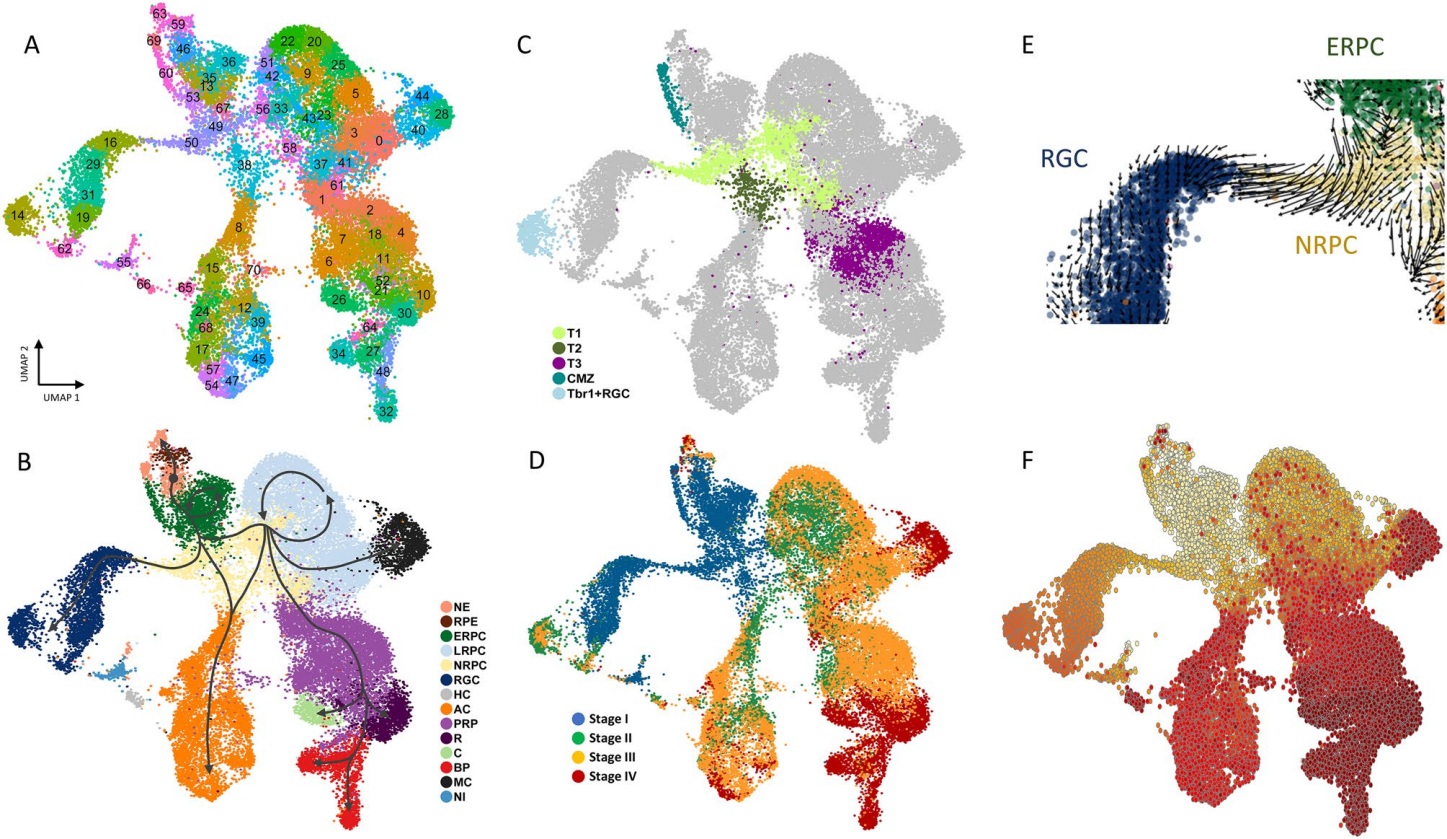
Joint scRNA-Seq-based UMAP of 38,091 cells corresponding to four developmental stages of native (NaR) and iPSC-derived retinal organoids (RO). (**A**) 2D UMAP manifold showing NaR and RO cells jointly and their assignment to 71 clusters by k-means clustering. (**B**) Merging the clusters in 13 major retinal cell types corresponding to neuroepithelium (NE), retinal pigmented epithelium (RPE), early (ERPC), late (LRPC), neurogenic retinal progenitor cells (NRPC), retinal ganglion cells (RGC), horizontal cells (HC), amacrine cells (AC), photoreceptor precursor cells (PRP), cones (C), rods (R), bipolar cells (BP), and Müller cells (MC), and cell-type not-identified (NI) on the basis of the expression of known marker genes (Fig. S2, Table S3). Information from temporal sampling, 3D UMAP manifolds (http://www.sig.hec.ulg.ac.be/giga), pseudo-time and RNA velocity analyses was combined and summarized graphically by the trajectories shown as dark grey arrows in (**B**). The grey dot within NE marks the origin or root of the trajectories. (**C**) Identifying known retinal sub-populations: post-mitotic transitional precursor cell populations (T1, T2, T3)^21^, Ciliary Marginal Zone (CMZ)^31^, and Tbr1 + retinal ganglionic cells (Trb1 + RGC)^32^. (**D**) Cells colored by developmental stage: I. blue = DD13 + E13, II. green = DD21 + P0, III. orange = DD25 + P5, IV. red = DD29 + P9. (**E**) Cell-specific RNA velocities^15^ confirming the ERPC → NRPC (T1) → RGC cellular trajectory. (**F**) Velocity-based diffusion pseudo-time analysis using a velocity-inferred transition matrix^14^. Increase in pseudo-time is marked by increase in redness.

We first analyzed NaR and RO data jointly to cover a maximum of intermediate developmental stages and hence generate the most continuous manifold possible. We used Canonical Correlation Analysis (CCA) implemented with Seurat^28^ to align the NaR and RO datasets based on the expression profiles of 1253 “most variable” genes (Table S2). We projected the corresponding 30-dimensional distances (based on the first 30 CCA) between cells in 2D- and 3D-space using Uniform Manifold Approximation and Projection (UMAP)^29^. We assigned all 38,091 cells jointly (NaR and RO) to 71 clusters by k-means clustering (Fig. 1A).

We defined gene expression signatures for 13 recognized retinal cell types using published information^8^ (Table S3 and Fig. S2), and regrouped the clusters accordingly in 13 cell types corresponding to neuroepithelium (NE), retinal pigmented epithelium (RPE), early (ERPC), late (LRPC), and neurogenic retinal progenitor cells (NRPC), retinal ganglion cells (RGC), horizontal cells (HC), amacrine cells (AC), photoreceptor precursor cells (PRP), cones (C), rods (R), bipolar cells (BP), and Müller cells (MC) (Fig. 1B). Using additional gene expression signatures we further identified: (i) actively dividing ERPC, LRPC and NRPC (in S and G2-M phases of the cell cycle)^30^, (ii) T1, T2 and T3 post-mitotic transitional precursor cell populations recognized in human native and hiPSC-derived retinae^21^, (iii) the ciliary marginal zone (CMZ)^31^, and (iv) a recently described subgroup of Tbr1^+^ RGC cells located in the inner plexiform layer^32^ (Fig. 1C, Fig. S2 and Table S3).

Labelling cells by developmental stages (stages I to IV, Fig. 1D) distinguished ERPC from LRPC, and revealed the expected sequence of emergence of RGC (stage I), followed by HC, AC and PRP (stage II and III), then C, R, BP and MC (stage III and IV). Cells assigned to the Tbr1 + RGC cluster appeared at stage II and III. T1, T2 and T3 cells appeared in that order. The UMAP manifold connected cell types consistently with known developmental trajectories^8,21,33,34^, including: (i) NE → RPE, (ii) NE → ERPC, (iii) ERPC → NRPC (T1) → RGC, (iv) LRPC → NRPC (T1 → T2) → AC, (v) LRPC → NRPC (T1) → PRP (T3) → C/R, and (vi) LRPC → MC. Reminiscent of previous studies^8,21^, the cluster of HC cells was disconnected from the rest of the manifold providing no information about their precursors. Cell-specific RNA velocities computed with *velocyto*^15^ were consistent with the ERPC → NRPC → RGC trajectory but otherwise difficult to interpret (Fig. 1E and Fig. S3). However, diffusion pseudotime analysis (using a velocity-inferred transition matrix) implemented with *scVelo*^14^ was remarkably proficient at ordering the four stages of development, as well as at identifying terminal cellular states (without benefitting from any information about development stage or root cells) (Fig. 1F).

### Comparison of NaR and RO cell fates in UMAP space highlights commonalities and differences in developmental trajectories

**Figure 2:**
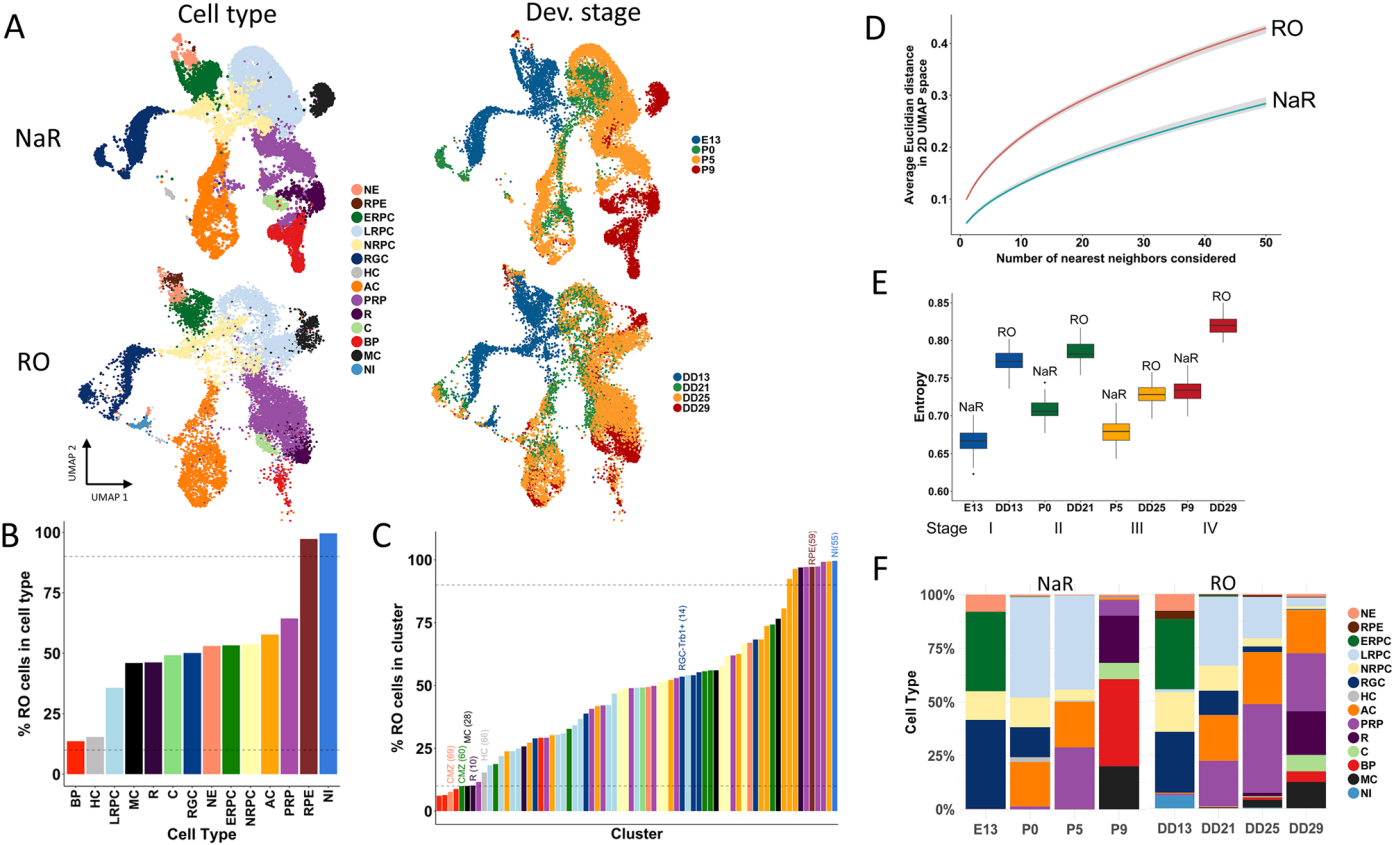
Comparison of NaR and RO cells in scRNA-Seq-based UMAP space. (**A**) Distribution of NaR (upper) versus RO (lower) cells across the UMAP manifold, sorted by cell type (left) and developmental stage (right). (**B**,**C**) Proportion of RO cells (adjusted for number of NaR and RO cells) in 13 cell types (**B**), and 71 clusters (**C**). 86% of cell types and 82% of clusters contain at least 10% of the least represented cell origin (NaR vs RO). Cell types are colored as in (**A**) and clusters are colored according to the cell type to which they were assigned. Notable clusters discussed in the main text are highlighted. (**D**) Larger average distance in 2D UMAP space (Y-axis) from *n* nearest neighbors (X-axis) for RO (red) than for NaR cells (blue). (**E**) Larger cell type diversity (sampling-based measure of entropy) in the four developmental stages for RO than for NaR. Developmental stages are colored-coded as in (**A**). (**F**) Proportions of cell types within a developmental stage for NaR (left) and RO (right).

We then focused on the comparison between NaR and RO cells. Global comparison of the distribution of NaR and RO cells across the manifold indicates that in vitro neuro-retinal differentiation from iPSCs largely recapitulates native development (Fig. 2A). This is substantiated by noting that 82% of the 71 clusters and 86% of the 13 cell types contain at least 10% of the least represented cell origin (NaR or RO) (Fig. 2B,C).

More granular examination, however, reveals noteworthy differences. The first is the apparent relaxation of pseudo-spatial and pseudo-temporal transcriptome control in RO versus NaR. Indeed, the developmental pathways traversed by NaR cells appear tighter than those of RO cells, while NaR cells sampled at a specific developmental stage seem to populate less cell types than RO cells. To quantify the former, we down-sampled cells to equalize NaR and RO cell numbers (within developmental stages) and computed the average distance from the *n* closest neighbors: it was highly significantly shorter for NaR than for RO (Fig. 2D). To quantify the latter, we measured the diversity of cell types within stages (using a measure of entropy): it was significantly lower in NaR than in RO for all four stages (Fig. 2E).

The second difference is the occurrence of NaR- or RO-specific clusters and cell types: (i) clusters corresponding to RPE cells are almost exclusively occupied by RO cells as a result of RPE elimination from NaR by dissection; (ii) the CMZ is absent in RO (only recently were culture conditions established for inducing selective CM retinal differentiation in human iPSC-derived organoids^35^; (iii) AC cluster 65 was only observed in RO); (iv) BP clusters 32, 34 and 48 are nearly exclusively composed of NaR cells, and (v) cluster 55 is exclusively populated by RO cells. It is thought to result from aberrant in vitro differentiation of NE into non-retinal neuronal cells. Indeed, it is connected to NE by a cellular bridge (Video: http://www.sig.hec.ulg.ac.be/giga), strongly expresses *Tbr1* and other genes typical of developing cortical neurons including reelin, without expressing the eye field transcription factors, *Pax6* and *Rax* (Tables S4 and S5).

The last noteworthy difference between both systems is the observation that PRP arise earlier in RO than in NaR and accumulate at the expense of other cell types (particularly LRPC), yet partially fail terminal differentiation particularly into BP cells (Fig. 2A,F).

### Retinal organoid in vitro culture conditions perturb genes and pathways that play key roles in native retinal development

**Figure 3:**
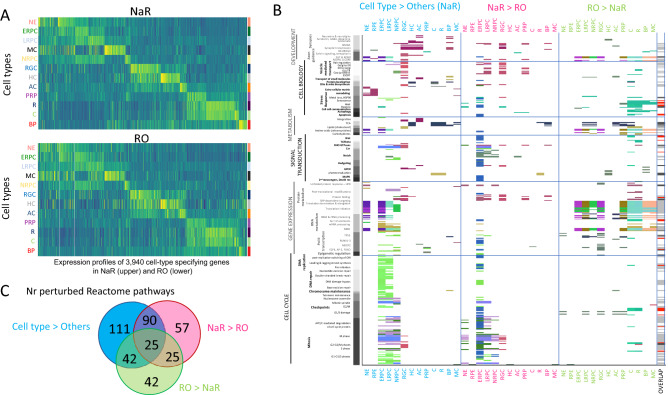
Comparison of the cell type-specific transcriptome of NaR and RO by means of scRNA-Seq. (**A**) Expression profiles in 13 cell types of 3940 genes that are dynamically regulated during in vivo retinal development (i.e. significantly overexpressed in at least one cell type when compared to all other ones in NaR) in NaR (upper panel) and RO (lower panel). Abbreviations refer to cell types and are as defined in Fig. 1. (**B**) Reactome pathways that are significantly (p  ≤ 0.001) enriched amongst differentially expressed genes (“Cell type > Others”: when comparing expression levels between specific cell types and all other cells in NaR; “NaR > RO” and “RO > NaR”: when comparing expression levels between NaR and RO cells within cell types). Reactome pathways (Y-axis) are sorted by “top level” pathway (cell cycle, gene expression, signal transduction, metabolism, cell biology and development) and sub-level therein. Colored tiles highlight the pathways that are significantly enriched in the corresponding comparisons (“Cell type > Others”, “NaR > RO” or “RO > NaR”). The colors of the tiles reflect similarity in the sets of Reactome “found entities” as described in the main text and Fig. S5. The last column (“Overlap”) shows the overlap between Reactome pathways enriched in cell type-specifying genes versus genes that are differentially expressed between NaR and RO: White: pathways significant in only one of the three comparisons, Black: pathways significantly different in all three comparisons, Grey: pathways significant in “Cell type > Others” and either “NaR > RO” or “RO > NaR”, Red: pathways significant in both “NaR > RO” and “RO > NaR”. (**C**) Number of unique and shared Reactome pathways between “Cell type > Others”, “NaR > RO” and “RO > NaR”. All overlaps are highly significant (p < 10^–6^).

To identify key genes for retinal differentiation, we performed differential expression analysis for each cell type against all others, first considering NaR cells only. We identified a total of 3940 genes with higher expression level in at least one of the 13 main cell-types compared to all other cell types merged (log-fold change ≥ 0.25 and p-value ≤ 0.001), hereafter referred to as “cell type-specifying” genes (Fig. 3A and Table S4). Of those, 3675 were also identified as dynamically regulated genes when using Monocle 2^17,36^ (Fig. S4 and Table S5).

We then searched for enriched Reactome pathways^37,38^ in the 13 lists of “cell type-specifying” genes. Two hundred sixty-eight pathways were significantly enriched (q-value ≤ 0.01) in at least one cell-type (Table S6). These corresponded primarily to (sorted by Reactome top-level pathway): (i) CELL CYCLE: accelerated cell division in ERPC, LRPC and NRPC, (ii) GENE EXPRESSION: intense post-transcriptional and translational activity in NE, ERPC, LRPC and NRPC, (iii) SIGNAL TRANSDUCTION: activation of RHO GTPase- and Notch-dependent signaling in ERPC, LRPC, NRPC, RGC, and LRPC, NRPC, respectively, as well as the GPCR-dependent phototransduction cascade in C and R, (iv) METABOLISM: activation of mitochondrial citric acid (TCA) cycle and respiratory electro transport in HC, C, R, BP, and MC, of cholesterol synthesis in ERPC and RGC, and of insulin- and glucagon-dependent metabolic integration in RGC and AC, (v) CELL BIOLOGY: enhanced remodelling of the extracellular matrix in NE, RPE and MC, and GAP junction trafficking in RGC, and (vi) DEVELOPMENT: activation of ROBO receptors-dependent axon guidance in NE, ERPC and LRPC, and of synapse formation in RGC, HC, AC and BP (Fig. 3B).

A Reactome pathway is considered enriched (in a list of submitted genes) if the number of genes in the list that are part of the pathway (the number of “found-entities”) is higher than expected by chance alone. The found-entities for different enriched Reactome pathways often show considerable overlap. As an example, the same five genes in the list of 465 ERPC-specifying genes explain the enrichment of both the “Leading strand synthesis” and “Polymerase switching” Reactome sub-pathways (found-entities: *Rfc5;Rfc4;Rfc1;Rfc2;Prim1*; Table S6). We devised a method to assign colors to enriched Reactome pathways such that pathways that were highlighted by strongly overlapping sets of found entities would have similar colors, while pathways highlighted by non-overlapping sets of found entities would have distinct colors (see Experimental procedures and Fig. S5). As an example, we can see from Fig. 3B that the 48 Reactome sub-pathways highlighted in NE correspond to six distinct sets of found entities or genes (six dominant colors), that one of these sets (bordeau) is also driving Reactome pathway enrichment in RPE, and that two others (indigo blue and purple) are also driving pathway enrichment in ERPC.

We then compared gene expression profiles between NaR and RO. At first sight, genes that were differentially expressed between cell-types in NaR appeared to recapitulate their in vivo expression profile quite well in RO (Fig. 3A). Yet, to better appreciate the differences between in vivo and in vitro retinal differentiation, we performed differential expression analysis between NaR and RO separately for each cell type. For each of the 13 major cell types, we generated two lists of genes corresponding respectively to those that were under-expressed in RO when compared to NaR (NaR > RO) and those that were over-expressed in RO when compared to NaR (RO > NaR) (q-value ≤ 0.01; Tables S7A and S7B). We then searched for biological pathways that were over-represented in the corresponding gene lists using Reactome. This yielded 197 downregulated (NaR > RO) and 134 upregulated (RO > NaR) pathways (Fig. 3B, Tables S8A and S8B). Strikingly, both down- and upregulated pathways (when comparing NaR and RO by cell type) exhibited considerable overlap with the pathways of cell-type specifying genes in NaR (Cell type > Others) (115/197 and 67/134, respectively; p-value < 10^–6^) (Fig. 3C). More specifically, (i) CELL CYCLE: the rate of cell division in NE, ERPC, LRPC and NRPC was reduced in RO when compared to NaR, (ii) GENE EXPRESSION: post-transcriptional and translational mechanisms were exacerbated in ERPC, LRPC, NRPC, RGC, PRP, C, R, BP and MC of RO, when compared to NaR, (iii) SIGNAL TRANSDUCTION: signal transduction via Wnt, TGF-beta, RHO GTPases, Esr, Notch, Hedgehog, MAPK, and Death receptors was diminished in RO when compared to NaR, particularly in ERPC and LRPC, while the phototransduction cascade was less active in RO-derived R than in NaR-derived R, (iv) METABOLISM: TCA cycle and respiratory electron transport was increased in RO’s LRPC, NRPC, AC, PRP and C (yet decreased in BP), cholesterol synthesis increased in RO’s C and R, and gluconeogenesis increased in RO’s PRP and R, (v) CELL BIOLOGY: stress response and apoptosis was reduced in RO’s ERPC, yet increased in RO’s C, R, BP and MC (i.e. at the latest stages of RO culture), and (vi) DEVELOPMENT: vesicle mediated transport and synapse formation was decreased in RO’s LRPC, RGC and PRP (Fig. 3B). As testified by their assigned colors in Fig. 3B, the found-entities driving Reactome pathway enrichment when analyzing cell-type specifying genes (Cell type > Others) or when comparing NaR and RO (NaR > RO and RO > NaR) showed considerable overlap (see also Fig. S5). Thus, the genes and pathways that appear to be the most perturbed by the RO in vitro culture conditions are also the ones that play key roles in NaR development.

### The expression level of many transcription factors is perturbed in retinal organoids

**Figure 4:**
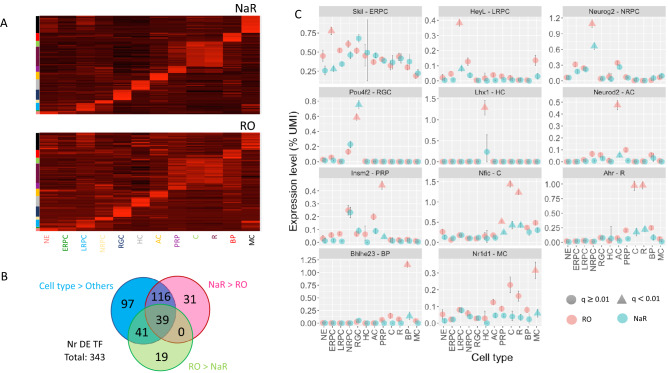
Comparison of the cell type-specific expression levels of TF in NaR and RO by means of scRNA-Seq. (**A**) Standardized expression levels of 107 most cell type-specific TF across 12 cell types in NaR (upper panel) and RO (lower panel). Abbreviations refer to cell types and are as defined in Fig. 1. (**B**) Number of differentially expressed TF in “Cell type > Others”, “NaR > RO”, and “RO > NaR”, with corresponding overlaps. The overlaps are highly significant (p < 10^–6^) assuming that TF are sampled randomly from the full collection of ~ 1500 TFs^39^. (**C**) Examples of TF that are (i) significantly overexpressed in one cell type when compared to all others in NaR, and (ii) significantly under- or over-expressed in that cell type between NaR and RO. The average expression levels (fraction of UMI) of the corresponding genes in the different cell types are shown for NaR (green) and RO (red). The error bars correspond to 99% confidence intervals determined by bootstrapping (n = 1000). Triangles mark cell types in which the corresponding gene is significantly (q < 0.01, i.e. accounting for multiple testing) overexpressed in NaR when compared to all other cell types combined (green), in which the expression level differs significantly (q < 0.01) between NaR and RO (red). The gene name and cell type of interest are given in the facet headers.

The 3940 cell type-specifying genes in NaR (Cell type > Others) comprised 293 transcription factors (TF)^39^, including 107 that were at least 1.5 times more strongly expressed in one cell type when compared to any of the other cell types (Fig. 4A and Table S4). The latter comprised 88 TF that were previously reported in the context of retinal development, as well as 19 novel ones (NE: *Peg3*; LRPC: *Lrrfip1*; MC: *Creb3l2*, *Csrnp1*, *Dbp, Nr4a1*, *Nr4a3*; HC: *Zfp618, Zfp804a*; AC: *Zfp503*; PRP: *Foxo3, Lcorl*; R*: Zfp516, Trps1, Ppard, Zc3h3*, *Mier1*, *Mier2, Lyar*; BP: *St18*) (Table S9). Of note, 16 of these (underlined) were also reported to be differentially expressed in developing mouse NaR by Clark et al.^8^. Contrary to the overall expression profile (Fig. 3A), visual examination of the expression profiles of the 107 most differentially expressed TF indicated considerable loss of cell-type specificity in RO (Fig. 4A). Indeed, 155 of the 293 (53%) differentially expressed TF were significantly (q < 0.01) under-expressed in RO when compared to NaR (at least in one cell type), while 80/293 (27%) were significantly (q < 0.01) over-expressed in RO when compared to NaR (Fig. 4B and Fig. S6). Striking examples include *Skil* (ERPC), *Heyl* (LRPC), *Neurog2* (NRPC), *Lhx1* (HC), *Neurod2* (AC), *Insm2* (PRP), *Nfic* (C/R), *Ahr* (C/R), *Bhlhe23* (BP) and *Nr1d1* (MC), which are all significantly under-expressed in RO when compared to NaR (Fig. 4C). An additional 31 TF (not part of the list of cell type-specifying genes) were down-regulated in RO, while 19 were upregulated (Fig. S6). Thus, the expression profile of a remarkably high number of TF appears perturbed in RO, and this may in part drive the differences observed between both systems, including with regards to Reactome pathways.

### Genes causing human retinal diseases are enriched in cell-type specifying genes

The RetNet database lists 281 human genes causing one of 27 retinal diseases in human^40^. We were able to interrogate the expression for 258 of corresponding murine orthologues using Cell Ranger (10× Genomics) (Table S10). Of these, 127 (= 49.2%) overlapped with our list of 3940 cell-type specifying genes. This is a significant enrichment (p-value < 10^–7^) when compared to the proportion of cell-type specifying genes amongst all genes expressed in NaR (3940/21,107 = 18.7%). Ten of the 127 were TF (= 7.9%), which is not significantly different (p-value = 0.86) from the proportion of TF amongst all cell-type specifying genes (293/3940 = 7.4%).

To evaluate for which human retinal disease-causing genes the mouse retinal organoid model might be applicable, we collected scRNA-Seq data for human fetal retinae at three developmental stages (FD59, FD82, FD125)^21^. We assigned individual cells to the same 13 cell types as done for the mouse. RGC and HC were abundant at FD59. AC, PRP, C, R, BP and MC appeared at FD82 and accounted for the majority of cells at FD125 (Fig. S7A). We then examined the correlation of the expression profiles (across the 13 cell types) for the 258 disease-causing genes between human native retinae, mouse native retinae and iPSC-derived mouse organoids. The correlation was highest for mouse NaR and mouse RO (median r = 0.82), followed by human and mouse NaR (median r = 0.35), and human NaR and mouse RO (median r = 0.34) (Fig. S7C). The correlation between human retina and mouse organoid was > 0.60 for 67 genes (26%). These were dominated by genes with significantly higher expression in PRP, C and/or R (44 genes) causing cone-rod dystrophy, retinitis pigmentosa, Bardet-Biedl syndrome, congenital stationary night blindness, Leber congenital amaurosis, and macular degeneration. The 67 genes also included BP-specifying genes (*Gpr179* and *Trpm1*) causing congenital stationary night blindness, MC-specifying genes (*Timp3* and *Clrn1*) causing respectively macular degeneration and retinitis pigmentosa/Usher syndrome, and ERPC/LRPC/NRPC-specifying genes (*Nr2f1*, *Kif11*, *Plk4*, *Nek2*) causing optic atrophy or syndromic retinopathy (Table S10).

## Discussion

We herein use scRNA-seq to compare the unfolding of the epigenetic program in in vivo versus in vitro (from iPSC) derived murine retinae at four matched development stages encompassing the presumed emergence times of the major retinal cell types (E13 vs DD13, P0 vs DD21, P5 vs DD25 and P9 vs DD29). Results obtained by combining information from (i) the analysis of four developmental stages, (ii) 3D UMAP manifolds visualized in virtual reality (http://www.sig.hec.ulg.ac.be/giga), and (iii) RNA velocity analysis, are in good agreement with the previously reported, main retinal developmental trajectories (Fig. 1). We identify ~ 4000 genes that are differentially expressed during in vivo retinal differentiation corresponding to tens of biological pathways pertaining to the cell cycle, gene expression, signal transduction, metabolism, cell biology and development (Fig. 3). Several of these pathways were previously highlighted when submitting differentially expressed genes identified from the analyses of bulk RNA-Seq data from multiple time points (E11 to P28) during retinal development^41^. Our data now allows to assign highlighted pathways to individual cell types. Differentially expressed genes include ~ 300 TF, of which ~ 100 are at least 1.5 times more strongly expressed in one specific retinal cell type when compared to all other ones. The latter include 19 TF not yet examined in detail in the field of retinal development which could serve as a starting point for functional investigations of the roles of these TF in retinogenesis and physiology.

We show that mouse retinal organoids broadly recapitulate the in vivo developmental program and trajectories even if developmental trajectories appear less canalized in RO when compared to NaR. We observed that PRP developed earlier and at the expense of other cell types’ terminal differentiation including BP (Fig. 2). This is certainly due at least in part to the culture condition aimed at promoting differentiation of photoreceptors^27^. We identify ~ 3000 genes that are differentially expressed between RO and NaR in at least one cell type, and the corresponding biological pathways pertaining in particular to (i) the rate of cell division which is reduced in RO RPCs when compared to NaR, (ii) post-transcriptional and translational mechanisms which appear exacerbated in the majority of RO cell types when compared to NaR, (iii) signal transduction via Wnt and Notch pathways which are diminished in RO RPCs when compared to NaR, (iv) the phototransduction cascade and synapse formation which appears reduced in RO, and, finally, (v) apoptosis and stress response which are increased at the latest stages of RO culture. Several of these perturbed pathways were highlighted before in analyses of bulk RNA-Seq data obtained during the development of NaR and RO^41^ and can now be assigned to cell type-specific transcriptome perturbations. Strikingly, the perturbed pathways show a highly significant overlap with those that were shown to be differentially expressed during in vivo development of NaR. We show that TF that are differentially expressed during in vivo retinal development are particularly sensitive to the iPSC culture conditions. This is likely to drive the perturbations of the above-mentioned biological pathways. Of interest, the list of TF whose expression appears perturbed in RO includes *Nfia* in BP, *Nfib* and *Nfix* in MC, as well as *Nfic* in C and R. Members of the Nuclear Factor I (NFI) family of transcription factors were recently shown to control cell-cycle exit as well cell fate specification of BP and MC^8^.

We finally show that genes that cause retinopathies in human are particularly enriched in genes that are significantly overexpressed in PRP, C and R, and identify a subset of disease-causing genes for which mouse iPSC-derived retinal organoids may be an appropriate model system on the basis of conserved expression profile between human retina and murine retinal organoids.

## Experimental procedures

### Generation of iPSC-derived retinal organoids

#### Maintenance of iPSCs

We used the mouse iPSC line generated from fibroblasts of C57BL/6 Nrl-eGFP transgenic mice^26^. The iPSCs were maintained in 60-mm Petri dishes (~ 0.6 × 10^5^ cells per dish) coated with 0.1% gelatin (G2625, Merck, Darmastadt, Germany) in Glasgow’s Minimum Essential Medium (GMEM, 11710035, Thermo Fisher Scientific (Fisher), Waltham, MA) supplemented with 10% Fetal Bovine Serum (FBS, 04-001-1, Biological Industries, Beit HaEmek, Israel), 1 mM sodium pyruvate (Merck), 0.1 mM MEM Non-Essential Amino Acids Solution (NEAA, Fisher), 0.1 mM 2-mercaptoethanol (2-ME, Wako Pure Chemical, Osaka, Japan), 100 U/mL penicillin–streptomycin (Fisher), 1000 U/mL of Leukemia inhibitory factor (Esgro LIF, Merck), 3 µM CHIR99021 (BioVision, Milpitas, CA) and 1 µM PD0325901 (Stemgent, Cambridge, MA).

#### Generation of iPSC-derived retinal organoids

Differentiation of iPSCs into retinal organoids was done using the SFEBq (serum-free floating culture of embryoid body-like organoids with quick re-aggregation) method according to^1^ with some modifications following^22,27^. The iPSCs were dissociated (DD0) after 4–5 days of maintenance using 0.25% trypsin/1 mM EDTA (Fisher) at 37 °C for 2 min. Embryoid body-like organoids were formed by adding 5000 cells/well in a low binding 96-well microplate (174925, Nunclon Sphera, Fisher) in 100 µL of differentiating medium. The differentiating medium was composed of GMEM, 0.1 mM AGN193109 (Toronto Research Chemicals, Toronto, Canada), 5% Knock-out Serum Replacement (Fisher), 1 mM Sodium Pyruvate, 0.1 mM NEAA and 0.1 mM 2-ME. At DD1, 20 µL of Matrigel Growth Factor Reduced Basement Matrix (Corning, Corning, NY) was added to obtain a final concentration equal to 2%. At DD8, retinal organoids were picked up and transferred in 60-mm Petri dishes with the maturation medium composed of Dulbecco’s Modified Eagle’s Medium/F-12 with glutamax (Fisher), 1% of N-2 supplement (Fisher) and 100 U/mL penicillin–streptomycin. Then, 0.5 µM retinoic acid (DD13 to DD18) (R2625, Merck), 1 mM of l-taurine (DD13 to DD29) (T8691, Merck) and 1% FBS (DD21 to DD29) were added to this maturation medium. Taurine and retinoic acid promote rod photoreceptor differentiation^27^. From DD8 to DD29, the cultures were maintained in a hyperoxic condition (37 °C, 40% O_2_/5% CO_2_). Development of retinal organoids was monitored using an EVOS FL digital inverted fluorescence microscope (Fisher) and GFP expression was confirmed from DD18.

### Ethical approval

All animal procedures were approved by the Animal Ethics Committee at University of Liège (approval no. 17-1908) and performed in accordance with the Guide for the Care and Use of Laboratory Animals at University of Liège which completely follow the recommendations in the ARRIVE guidelines.

### Immunofluorescence

Retinal organoids were fixed for 20 min at room temperature in 4% paraformaldehyde (PFA) in phosphate saline (PBS) at pH 7.4. They were equilibrated overnight in 30% sucrose (in PBS) at 4 °C before cryoprotection. Eyeballs from wild type C57BL/6 mice, used as positive controls, were enucleated and punctured in the center of the cornea before fixation for 1 h in 4% PFA and at room temperature, then washed in PBS and incubated in 30% sucrose at 4 °C overnight. The samples were embedded in Richard-Allan Scientific NEG-50 Frozen Section medium (Fisher). Slices of 10 to 15 µm were generated with a cryostat and placed on Superfrost Ultra Plus slides (Fisher). For immunofluorescence, slides were first incubated in Blocking One solution (Nacalai Tesque, Kyoto, Japan) for 1 h at room temperature, then at 4 °C overnight with primary antibodies diluted in Dako REAL Antibody Diluent (Agilent, Santa Clara, CA). We used the following primary antibodies against: Protein Kinase Cα diluted at 1:500 (Antibody Registry ID: AB_477345, Merck), Recoverin at 1:1000 (AB_2253622, Merck), Calretinin at 1:500 (AB_2313763, Swant, Marly, Switzerland), Pax6 at 1:100 (AB_2313780, BioLegend, San Diego, CA), Rhodopsin at 1:1000 (RET-P1, AB_260838, Merck), Chx10 at 1:1000 (AB_2314191, Exalpha Biologicals, Shirley, MA). After 24 h incubation, slides were washed three times for 5 min in PBS/0.05% Tween then incubated with appropriate secondary antibodies in the dark at room temperature (anti- rabbit IgG-AF488 and -AF647, anti-mouse IgG-AF555 and anti-sheep IgG-AF555, all from Fisher) and 1:1000 4′,6-diamidino-2-phenylindole (DAPI) in Dako REAL Antibody Diluent. After another wash in PBS/0.05% Tween, slides were mounted with FluorSave Reagent (Merck). Images were taken with a Nikon Eclipse T_i_ confocal microscope.

### Single cell RNA-Seq

#### Dissociation of native retinal tissue and 3D-culture retinal organoids

The dissociation of mouse retinae and 3D retinal organoids was inspired by the protocol of^9^. Eyeballs of C57BL/6 wild type mice were enucleated at time points E13, P0, P5 and P9. Dissected retinae were placed in Dulbecco’s Phosphate Buffered Saline (DPBS, Fisher). Optic vesicle (OV)-like structures of the iPSCs derived 3D retinal organoids were dissected at DD13, DD21, DD25 and DD29 and transferred in DPBS as well. Papain at final concentration 4 U/mL (Worthington Biochemical Corporation, Lakewood, NJ) was added to the samples and incubated at 37 °C for 45 and 30 min, respectively. To stop the reaction, 0.15% ovomucoid (Worthington Biochemical Corporation) was added. Samples were then centrifuged in order to eliminate the supernatant and cells were resuspended in DPBS. Cell numbers and proportion of living cells were estimated by a Trypan Blue staining using a Countess II cell counter (Fisher).

#### scRNA-Seq

We generated two biological replicate samples (NaR and RO) for stages I to III and one biological replicate for stage IV. We loaded ~ 15,700 cells for biological replicate 1 (stage I–IV) and ~ 10,000 cells for biological replicate 2 (stage I–III) in a Chromium Controller instrument (10× Genomics, Pleasanton, CA). Sequencing libraries were generated using Chromium Single Cell 3′ reagent kits v2.0 following the recommendations of the manufacturer (10× Genomics). Sequencing was conducted on an Illumina NextSeq 500 instrument (Illumina, San Diego, CA).

#### Bioinformatic analyses

Demultiplexing, alignment, filtering, barcode counting, UMI counting, and aggregation of multiple runs were conducted using Cell Ranger v2.1.1 (10× Genomics). Further filtering, k-means clustering, and UMAP projection were conducted using Seurat software suite (https://satijalab.org/seurat/)^28^. Velocity analysis was performed using the Velocyto R^15^ and scVelo^14^ packages. Single-cell trajectory inference and pseudotime analyses were conducted using Monocle2 (http://cole-trapnell-lab.github.io/monocle-release/)^17^.

### Downstream analyses

#### Width of developmental trajectories in 2D UMAP space

To test whether the developmental trajectories were more tightly regulated in NaR than in RO, we computed the average distance (computed as the Euclidian distance in 2D-UMAP space, i.e.$\sqrt{{({x}_{1}-{x}_{2})}^{2}+{({y}_{1}-{y}_{2})}^{2}}$) between 500 randomly selected NaR and 500 randomly selected RO cells and their *n* nearest neighbors (with *n* ranging from 1 to 50). The number of cells per developmental stage was adjusted between NaR and RO by down sampling to the number of the least populated source. The corresponding calculations were performed five times. The curves shown in Fig. 2D correspond to the averages across the five replicates. The grey confidence zone in Fig. 2D is bounded by the maxima and minima across the five replicates. The corresponding script was written in Perl (Dev_path_width.pl) and the graph generated in R (Path_width.R).

#### Within developmental stage cell type entropy

To compare cell type diversity within developmental stage between NaR and RO, we first equalized the number of cells within developmental stages between NaR and RO by randomly dropping cells from the most populated source. We then sampled two cells within cell source (NaR and RO) and developmental stage and checked whether they were from the same cell type or not. This was repeated 1000 times yielding a measure of cell type diversity akin to (1-Entropy). Down-sampling of cells was repeated 100 times. Each data point in Fig. 2E corresponds to (1-Entropy) for one such random sample. The corresponding script was written in Perl (entropy.pl) and the graph generated in R (Entropy.R).

#### Differential expression analyses

Differential expression analyses to identify genes that are upregulated in specific cell types when compared to all other ones (Cell type > Others) or that are differentially expressed between NaR and RO in a given cell type (NaR > RO and RO > NaR) were performed with the *Findmarkers* function in Seurat (https://satijalab.org/seurat/)^28^.

#### Pathway analyses

Pathway enrichment analyses were conducted using the on-line Reactome analysis tools^37,38^. Mouse gene identifiers were converted to human counterparts. Pathway analysis results were downloaded as flat files. A total of 392 pathways with enrichment p-value ≤ 0.01 in at least one analysis were kept and manually sorted according to Reactome hierarchy (Man_processed_reactome_output.txt). A pathway is enriched in a list of genes if it contains more components of the pathway than expected by chance (given the number of genes in the list). The overlapping genes (“found entities”) hence define the enrichment. The same pathway can be enriched in two gene lists due to the same, distinct or partially overlapping sets of “found entities”. We quantified the degree of overlap between sets of “found entities” for the 1313 pathway enrichments using principal component (PC) analysis in a space defined by the presence/absence of 1335 genes. The distance between sets of “found entities” in a space consisting of the 20 first PCs was projected in 3D space using t-distributed stochastic neighbor embedding (tSNE) implemented with the *Rtsne* R function^42^. 3D tSNE coordinates were converted to hexadecimal RGB codes and used to color the sets of “found entities” (corresponding to the enrichment of a pathway in a specific gene list) when generating 2D tSNE graphs (Fig. S4), or when generating a tile showing the pathways enriched in specific analyses (Cell type > Others, NaR > RO or RO > NaR) and cell types within the analysis (NE, RPE, ERPC, LRPC, NRPC, RGC, HC, AC, PRP, C, R, BP or MC) (Fig. 3B). The corresponding scripts were written in Perl (Reactome_analysis.pl) and R (Reactome_analysis.R).

## Supplementary Information

Supplementary Figures.

Supplementary Tables.

## Data Availability

All data generated as part of this work are available without restriction. scRNA-Seq have been deposited (Array Express) under accession number E-MTAB-9395 (https://www.ebi.ac.uk/biostudies/arrayexpress/studies/E-MTAB-9395). Other data and analysis pipelines are available at https://doi.org/10.5281/zenodo.7486356. Human data used in this study are available from Gene Expression Omnibus (accession nr GSE142526), and the Retinal Information Network or RetNet (https://sph.uth.edu/retnet/).
