# Supplementary material for: Comparing the transcriptome of developing native and iPSC-derived mouse retinae by single cell RNA sequencing
Source: Sci Rep. 2023 Jan 21;13:1223. doi: 10.1038/s41598-023-28429-y (PMC9867755; doi:10.1038/s41598-023-28429-y)
Supplement: Supplementary file 1 — Supplementary Figures. [file 41598_2023_28429_MOESM1_ESM.docx]

**Supplemental Information**

**Comparing the transcriptome of developing native and iPS-derived murine retinae by single cell RNA sequencing**

Anouk Georges,^1,2^ Arnaud Lavergne,^3^ Michiko Mandai,^4^ Fanny Lepiemme,^1^ Latifa Karim,^5^ Loic Demeulenaere,^6^ Diego Aguilar,^6^ Michael Schyns,^7^ Laurent Nguyen,^1^ Jean-Marie Rakic,^2^ Masayo Takahashi,^8^ Michel Georges,^6,^* and Haruko Takeda^6^

^1^GIGA Stem Cells, GIGA Institute, University of Liège, Belgium. ^2^Department of Ophthalmology, Faculty of Medicine and CHU University Hospital, University of Liège, Belgium. ^3^GIGA Bioinformatics platform, GIGA Institute, University of Liège, Belgium. ^4^Laboratory for Retinal Regeneration, Center for Developmental Biology, RIKEN, Japan. ^5^GIGA Genomics Platform, GIGA Institute, University of Liège, Belgium. ^6^Unit of Animal Genomics, GIGA Institute, University of Liège, Belgium. ^7^Digital Business, HEC Management School, University of Liège, Belgium. ^8^Laboratory for Retinal Regeneration, Center for Biosystems Dynamics Research, RIKEN, Japan.

*Correspondence: michel.georges@uliege.be

## Figure S1:

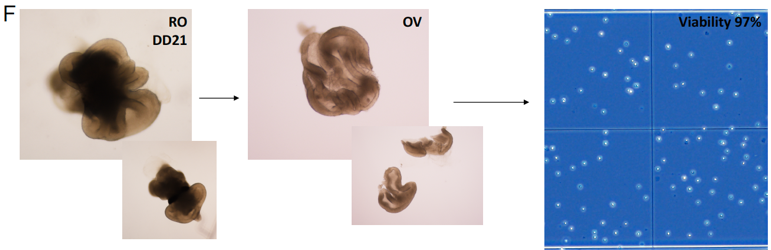


**Figure S1. (A - C) Expected layered expression of cell-type specific immunohistochemical markers.** Immunohistochemical markers of cellular subtypes in Nrl-GFP iPSC-derived RO (DD21 and DD28) and NaR (E13 and P9). **(A)** Photoreceptor cells**:** Nrl-GFP (green), Recoverin (white) and Rhodopsin (red) specify rod photoreceptor cells; DAPI (blue) marks nuclei of all cells. INL, inner nuclear layer; ONL, outer nuclear layer; GCL, ganglion cell layer. **(B)** Bipolar cells: Chx10 (red) and PKC-α (white) are markers for bipolar cells. Nrl-GFP (green), DAPI (blue), INL, ONL, GCL, as above. **(C)** Retinal ganglion cells: Pax6 (white) and Isl1 (magenta) are markers for retinal ganglion cells. DAPI (blue) as above. The white arrows show Isl1 positive retinal ganglion cells in RO. Calretinin (white) is a marker for inner retinal cells (retinal ganglion cells and amacrine cells). Chx10 (red) and DAPI (blue) as above. Scale bars, 50 µm for all. **(D**) **Schematic of anatomy of retinal layers. (E)** **Three-dimensional *in vitro* differentiation of iPSC-derived retinal organoids (RO).** Morphology of iPSCs five days post-thawing (miPSCs d5). Cells in undifferentiated state are circumferential and “domed shaped”, and surrounded by a luminous halo (white arrow). Some unstable colonies tend to differentiate with a fibroblastic morphology instead of round shape (red arrow). Morphology of RO from differentiation DD1 to DD28 following the modified SFEBq protocol (Assawachananont et al., 2014; Osakada et al., 2008). DD1, rapid re-aggregation of iPSCs after dissociation and passage in a 96-well plate; DD5, appearance of retinal neuro-epithelium on the retinal aggregates (light edges); DD7, evagination of retinal neuro-epithelium; DD8-DD20, growing of evaginating OV-like structures; DD22-DD28, GFP expression under control of the *Nrl* promotor in the photoreceptor layer of retinal organoids. Scale bars: 400 µm for d5; 1000 µm for others. **(F) Dissection of OV-like structures and dissociation of cells into a viable single cell solution.** Manual dissection of RO at stage II (DD21). The retinal neuro-epithelium tissue was isolated from the pigmented inner cell mass and dissociated into a homogenous solution of single cells. Viability of cells in the solution was 97%. Concentration of cells was 2.0 x 10^6^ cells/ml.

## Figure S2:

**
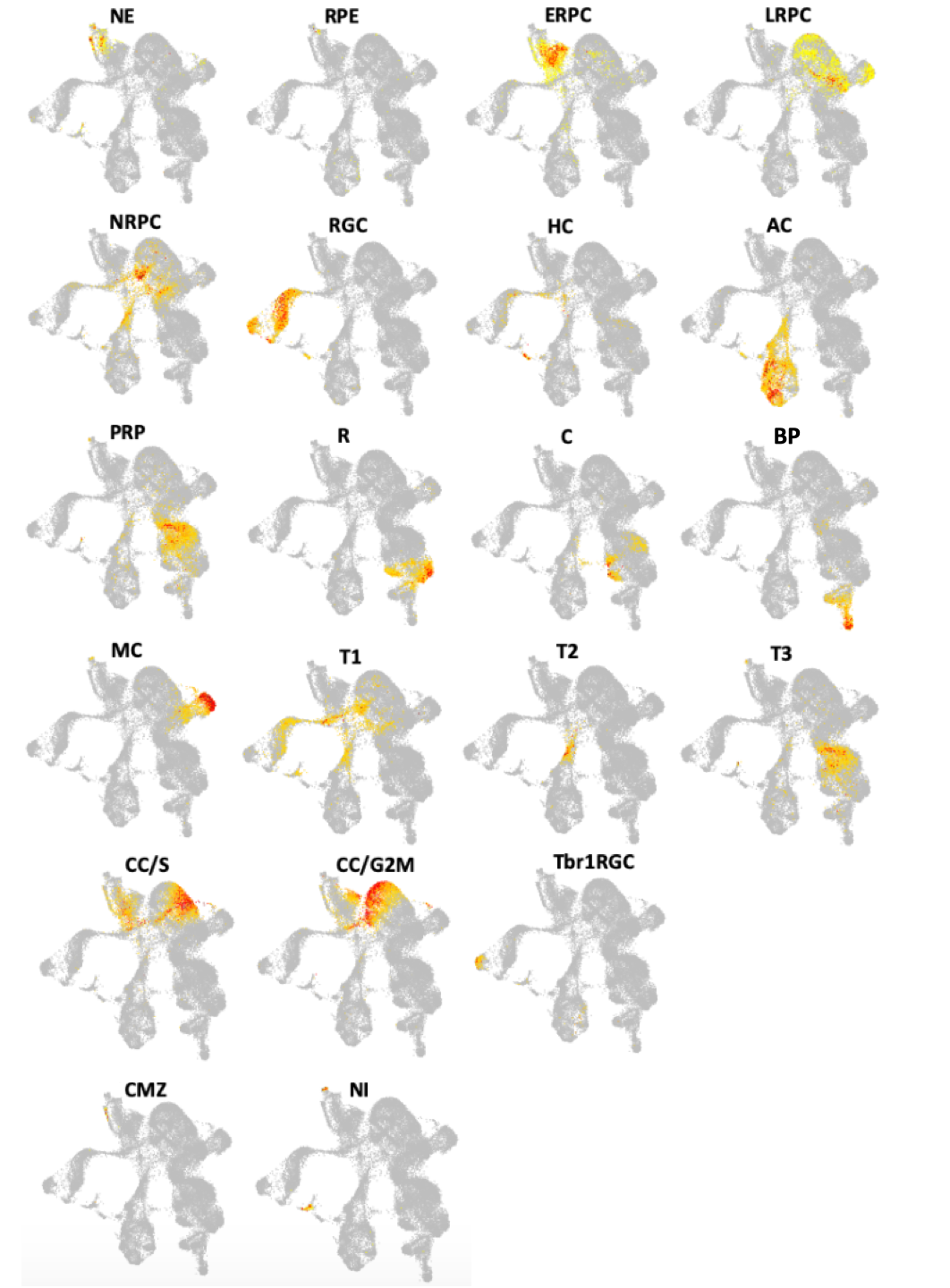
**

**Figure S2.** UMAP manifolds showing gene expression signatures for (i) 13 previously recognized major retinal cell types: NE, RPE, ERPC, LRPC, NRPC, RGC, HC, AC, PRP, R, C, BP and MC, and for (ii) previously recognized subpopulations: transitional post-mitotic progenitor subpopulations T1, T2 and T3 (Sridhar et al., 2020), Tbr1 positive RGC (Liu et al., 2018), dividing cells in S (CC/S) or G2-M (CC/G2M) cell cycle phase (Tirosh et al., 2016), the ciliary marginal zone (CMZ) (Trimarchi et al., 2009), and (iii) a non-identified population (NI) of non-retinal neuronal cells found only in RO.

**Figure S3:**

**Figure S3:** RNA velocities computed with velocyto (La Manno et al. 2018).

## Figure S4:


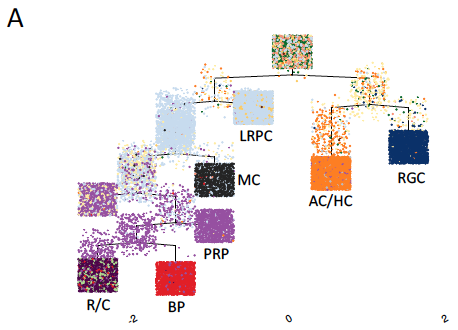

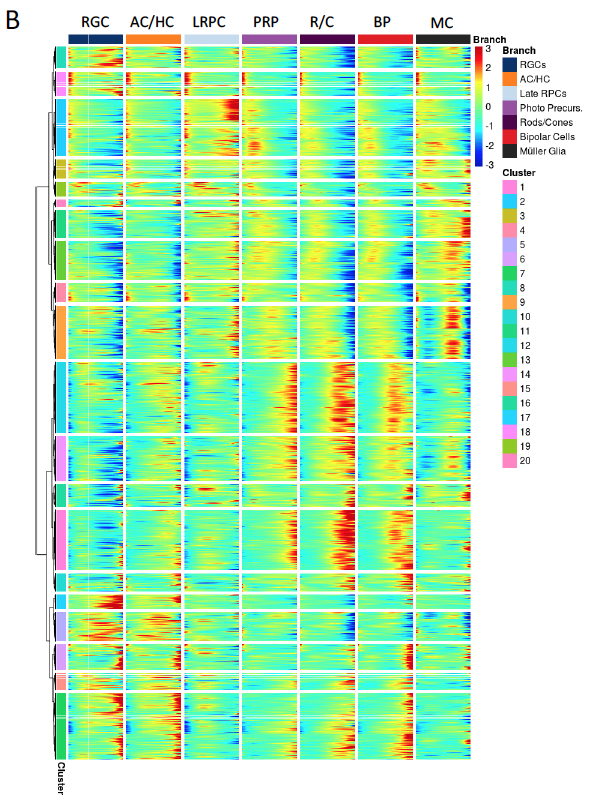


**Figure S4. (A)** Developmental trajectory obtained without supervision with Monocle 2 (Trapnell et al., 2014) using NaR cells only. Colors correspond to cell types as defined in the main text and color-coded as in the main figures. As expected, terminal leaves correspond to terminally differentiated cell types (from left to right: R/C, BP, MC, AC/HC and RGC) or to niches of progenitor cells (LRPC, PRP). **(B)** Expression levels (in pseudo-color) along the seven branches of the Monocle 2 tree (X axis) of 10,269 genes that are dynamically regulated as a function of pseudo-time (q < 0.001) and grouped in 20 clusters according to their expression pattern (Y axis).

## Figure S5:

**Figure S5. (A)** tSNE map (dimensions 1 and 2 of 3) of 1,313 gene sets (“found entities”) marking enriched Reactome pathways (each little circle corresponding to a tile in Figure 3B). Overlapping sets (in terms of gene content) are close to each other in tSNE space (3D). The gene sets are colored in RGB scale where tSNE dimension 1 (tSNE1) determines the intensity of red, tSNE2 the intensity of green, and tSNE3 the intensity of blue. **(B)** Same data points as for (A) but colored by contrast of origin: “Cell type > OTHERS”: turquoise; “NaR > RO”: magenta; “RO > NaR”: lime. One can see that many clusters encompass gene sets corresponding to distinct contrasts, hence highlighting the strong overlap between Reactome pathways that are essential for normal retinal development (“Cell type > OTHERS”), and those that are perturbed in RO relative to NaR (“NaR > RO” and “RO > NaR”).

## Figure S6:

**Figure S6. (A)** Transcription factors (TF) that are (i) differentially regulated between cell types in NaR (Cell type > others, turquoise), (ii) under-expressed in RO when compared to NaR (NaR > RO, magenta), or (iii) over-expressed in RO when compared to NaR (RO > NaR, lime). OVERLAP: TF that are differentially expressed in the three conditions (Cell type > others, NaR > RO and RO > NaR) are marked in black. TF that are differentially expressed during retinal development (Cell type > others) and in one of the NaR vs RO conditions (NaR > RO or RO > NaR) are marked in grey. Acronyms for cell types are as in the remainder of the manuscript. **(B)** Average expression level (% UMI) in 12 retinal cell types in NaR (green) and RO (red) of the 343 TF (cfr. A) that are significantly overexpressed in at least one cell type when compared to all others in NaR (293) and/or significantly over- or under-expressed in RO when compared to NaR in at least one cell type. Green triangles mark cell types in which the corresponding gene is significantly (q < 0.01, i.e. accounting for multiple testing) overexpressed in NaR when compared to all other cell types combined. Red triangles mark cell types in which the expression level differs significantly (q < 0.01) between NaR and RO. The gene names are given in the facet headers. The cell types in which the TF is overexpressed (NaR) are indicated by the colored bars (color code as in A and other figures). Red asterisks mark the genes that were selected for Figure 4C.

## Figure S7:

A B


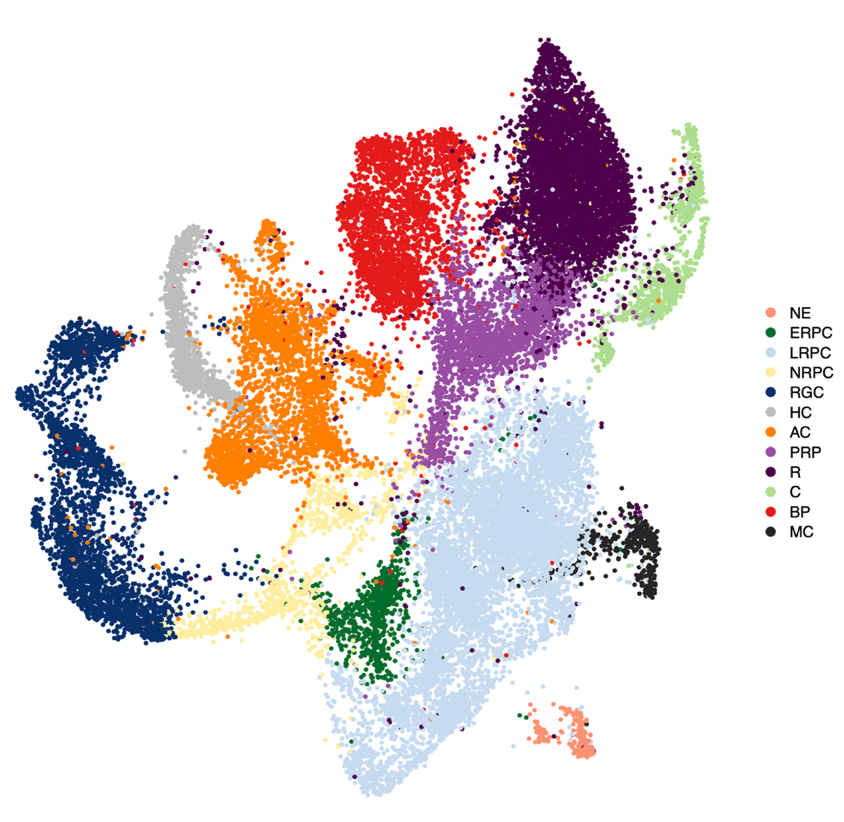


C


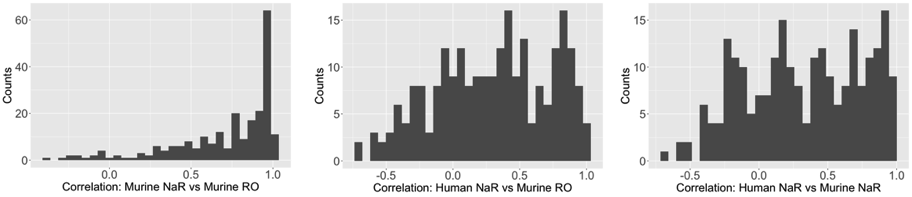


**Figure S7. (A)** UMAP of scRNA-Seq data of human fetal retina (FD59, FD82 and FD125) obtained with data from Sridhar *et al.* (2020). Cells were clustered and assigned to specific cell types following the same procedure as used for the murine data. **(B)** Proportion of the different cell types at the three different developmental stages (D59, D82c and D125c; labelled following Sridhar et al.). **(C)** Correlation between the expression profiles of 258 genes causing human retinopathies in human native retina (Human NaR), murine native retina (Murine NaR) and murine retinal organoids (Murine RO).
